# The Effect of *Dioscoreae Rhizoma* on Gastrointestinal Function: A Systematic Review

**DOI:** 10.3390/nu17182943

**Published:** 2025-09-12

**Authors:** Ji-Hye Lee, So-Young Park, Min-Seok Jo, Jae-Woo Park, Jinsung Kim, Seok-Jae Ko

**Affiliations:** 1 Department of Clinical Korean Medicine, Graduate School, Kyung Hee University, Seoul 02447, Republic of Korea; jhfuture0@naver.com (J.-H.L.); thebestbrain@naver.com (M.-S.J.); pjw2907@khu.ac.kr (J.-W.P.); oridoc@khu.ac.kr (J.K.); 2 Department of Clinical Korean Medicine, Sangji University, Wonju 26339, Republic of Korea; parksoyoung02180@gmail.com; 3 Department of Digestive Diseases, College of Korean Medicine, Kyung Hee University, Seoul 02447, Republic of Korea; 4 Department of Korean Internal Medicine, Kyung Hee University College of Korean Medicine, Kyung Hee University Hospital at Gangdong, Seoul 02447, Republic of Korea; 5 Division of Digestive Diseases, Department of Korean Internal Medicine, Kyung Hee University Korean Medicine Hospital, Seoul 02447, Republic of Korea

**Keywords:** *Dioscoreae Rhizoma*, yam, gastrointestinal, systematic review

## Abstract

**Background/Objectives**: *Dioscoreae Rhizoma*, commonly known as yam, has long been used in East Asia as a medicinal food for gastrointestinal (GI) health. This systematic review aimed to assess the GI-related benefits of *Dioscoreae Rhizoma* by synthesizing findings from both human clinical trials and in vivo experimental studies. **Methods**: A structured search of eight major databases—including PubMed, EMBASE, and Web of Science—was conducted through April 2025. This systematic review includes both human and in vivo animal studies that investigated the effects of *Dioscoreae Rhizoma* on gastrointestinal function. Studies such as in vitro experiments, non-original articles and studies involving multi-herbal formulations were excluded. Risk of bias was assessed with three different tools including the Cochrane Risk of Bias 2 (RoB 2) tool. **Results**: Twenty-seven studies met the inclusion criteria, comprising two human trials and twenty-five animal experiments. Clinical trials reported improvements in gut-microbiota balance, glycemic control, and postsurgical recovery, including enhanced wound healing and reduced infection rates. In animal models, yam-derived interventions attenuated inflammatory responses, enhanced antioxidant defenses, preserved mucosal-barrier integrity, and favorably modified gut-microbiota composition. **Discussion and Conclusions**: Accumulating evidence supports the GI-beneficial effects of *Dioscoreae Rhizoma*, mediated through diverse biological pathways, including immunomodulation, antioxidation, and microbiota regulation. This study has limitations on lack of high-quality human studies, small sample size and heterogeneity among studies regarding different plant parts used, extraction processes, and dosage. Further rigorously designed studies are warranted to clarify the mechanisms, standardize intervention protocols, and validate clinical efficacy.

## 1. Introduction

In recent years, food-based interventions that use traditional medicinal plants have attracted increasing attention as adjunct strategies for preventing and treating gastrointestinal diseases [1]. In East Asia, *Dioscoreae Rhizoma*, commonly known as yam, has long been consumed as a medicinal-cum-food ingredient that supports gastrointestinal health and immune regulation [2]. It contains resistant starch, soluble fiber, diosgenin, complex polysaccharides, and polyphenols that exhibit antioxidant, anti-inflammatory, glucose-modulating, and tissue-regenerative activities [3]. *Dioscoreae Rhizoma* is particularly beneficial for colorectal cancer (CRC) patients with type 2 diabetes mellitus (T2DM), whose hyperglycemia delays wound closure, increases infection risk, and prolongs hospital stays, making postoperative nutrition even more critical [4,5,6,7].

This study aims to investigate the gastrointestinal effects of *Dioscoreae Rhizoma* through a systematic and comprehensive evaluation of human clinical trials and in vivo studies. We aimed to classify the observed outcomes into functional domains and correlate them with relevant biomarkers, thereby elucidating the pathways through which *Dioscoreae Rhizoma* exerts its anti-inflammatory, antioxidant, microbiota-modulating, and mucosal-protective effects on gastrointestinal function.

## 2. Materials and Methods

This study comprehensively reviewed human and animal studies published up to April 2025 that investigated the effects of *Dioscoreae Rhizoma* on gastrointestinal function. We categorized the observed outcomes into key physiological domains, including gastrointestinal motility, inflammation, immune modulation, gut microbiota composition, and mucosal protection. Additionally, we synthesized integrative evidence supporting the therapeutic potential of *Dioscoreae Rhizoma* in the management of gastrointestinal disorders.

The methodology adhered to the Preferred Reporting Items for Systematic Reviews and Meta-Analyses (PRISMA) 2020 guidelines [8]. The protocol was registered on the Open Science Framework under the title “The effect of *Dioscoreae Rhizoma* on gastrointestinal function: A systematic review” (https://doi.org/10.17605/OSF.IO/WJM23, accessed on 19 May 2025).

### 2.1. Search Strategy

A standardized search strategy was used to retrieve relevant human clinical trials and in vivo animal studies that evaluated the effects of *Dioscoreae Rhizoma* on gastrointestinal function. Search terms encompassed gastrointestinal-related keywords—such as “dyspepsia,” “indigestion,” “digestion,” “gastro,” “epigastric,” “stomach,” “gastrointestinal,” “gastr,” “intestin,” “colon,” “bowel,” “colitis,” and “Crohn”—in combination with *Dioscorea*-related terms, including “*Dioscorea*” and “yam.”

Eight electronic databases were systematically searched: PubMed, EMBASE, Web of Science, the Cochrane Library, China National Knowledge Infrastructure (CNKI), Citation Information by NII (CiNii), the Allied and Complementary Medicine Database (AMED), and the Oriental Medicine Advanced Searching Integrated System (OASIS). Search strategies were adapted to each database’s structure and indexing conventions; for example, topic- or abstract-specific filters were applied in PubMed, EMBASE, and Web of Science.

The search covered all years available within each database up to April 2025. Detailed search strings and strategies for PubMed are presented in Table 1.

### 2.2. Selection Criteria

Study selection followed predetermined inclusion and exclusion criteria (Table 2).

#### 2.2.1. Inclusion Criteria

This systematic review incorporated both human and in vivo animal studies that investigated the effects of *Dioscoreae Rhizoma* on gastrointestinal function. Eligible studies were required to evaluate outcomes related to gastrointestinal motility, inflammation, mucosal protection, immune modulation, or gut-microbiota composition. Disease models commonly associated with gastrointestinal dysfunction—such as colitis, gastric ulcer, colon cancer, and antibiotic-associated diarrhea—were included. Both healthy and disease-induced models were eligible, provided that gastrointestinal outcomes were clearly defined and the independent effects of *Dioscoreae Rhizoma* or its derivatives could be evaluated.

#### 2.2.2. Exclusion Criteria

Studies were excluded if they met any of the following criteria: (1) in vitro experiments using cell cultures or tissue samples; (2) studies involving multi-herbal formulations in which the independent effect of *Dioscoreae Rhizoma* could not be isolated; (3) non-original articles, including narrative reviews, case reports, case series, editorials, or conference abstracts lacking full methodological transparency; and (4) studies for which the full text was unavailable, precluding quality assessment and data extraction.

### 2.3. Data Extraction

Two reviewers (J.-H.L. and S.-Y.P.) independently screened studies for eligibility in accordance with PRISMA 2020. Screening proceeded in three stages: title screening, abstract screening, and full-text assessment. At each stage, the inclusion and exclusion criteria were rigorously applied. All records were managed with EndNote 21 (Clarivate, Philadelphia, PA, USA) to facilitate systematic organization and removal of duplicates.

For the final set of included studies, data were collected with a pre-established extraction template. For in vivo animal studies, the following details were recorded: year of publication, first author, disease model, species, experimental inducer, route and duration of administration, proposed mechanisms, primary outcomes, and reported efficacy. For human clinical studies, data fields included study objectives, intervention details, participant numbers, intervention duration, analytical methods, key outcomes, and conclusions.

Discrepancies between the two primary reviewers were resolved through discussion; unresolved disagreements were adjudicated by a third reviewer (S.-J.K.).

### 2.4. Quality Control

To ensure methodological rigor, the risk of bias was independently evaluated for human and in vivo animal studies using tools appropriate to each design.

For randomized human studies, risk of bias was assessed with the Cochrane Risk of Bias 2 (RoB 2) tool [9]. Two independent reviewers (J.-H.L. and S.-Y.P.) evaluated five domains: (1) bias arising from the randomization process; (2) bias due to deviations from intended interventions; (3) bias due to missing outcome data; (4) bias in measurement of the outcome; and (5) bias in selection of the reported result. Each domain was rated as “low risk,” “some concerns,” or “high risk.”

For non-randomized human studies, risk of bias was assessed with the Risk of Bias in Non-randomized Studies of Interventions Version 2 (ROBINS-I V2) tool [10]. Seven domains were evaluated: (1) bias due to confounding; (2) bias in classification of interventions; (3) bias in participant selection; (4) bias due to deviations from intended interventions; (5) bias due to missing data; (6) bias in measurement of the outcome; and (7) bias in selection of the reported result. Each domain was rated as “low,” “moderate,” “serious,” or “critical” risk of bias. Discrepancies were resolved by consensus or consultation with a third reviewer (S.-J.K.).

For in vivo animal studies, risk of bias was assessed with the Systematic Review Centre for Laboratory Animal Experimentation (SYRCLE) Risk of Bias tool [11], which addresses ten domains: (1) sequence generation; (2) baseline characteristics; (3) allocation concealment; (4) random housing; (5) blinding of caregivers and investigators; (6) random outcome assessment; (7) blinding of outcome assessors; (8) incomplete outcome data; (9) selective outcome reporting; and (10) other potential sources of bias. Each domain was rated as “low risk,” “some concerns,” “high risk,” or “no information.”

Conflicts between reviewers were discussed to reach agreement; unresolved issues were adjudicated by a third reviewer (S.-J.K.), who served as the final arbiter.

## 3. Results

### 3.1. Search Process for Included Studies

An initial yield of 865 records was obtained through extensive searches across eight electronic databases: Web of Science, PubMed, CNKI, CiNii, EMBASE, Cochrane Library, OASIS, and AMED. After removing 296 duplicate records, 569 entries remained for title and abstract screening. Of these, 498 were excluded as unrelated to the topic.

Subsequently, 71 articles were selected for full-text retrieval. Four reports could not be retrieved. The remaining 67 articles underwent full-text evaluation for eligibility. Based on predefined criteria, 8 studies using herbal mixtures (in which the independent effect of *Dioscoreae Rhizoma* could not be determined), 25 in vitro studies, 5 non-original articles, and 2 studies on non-targeted diseases were excluded.

**Figure 1:**
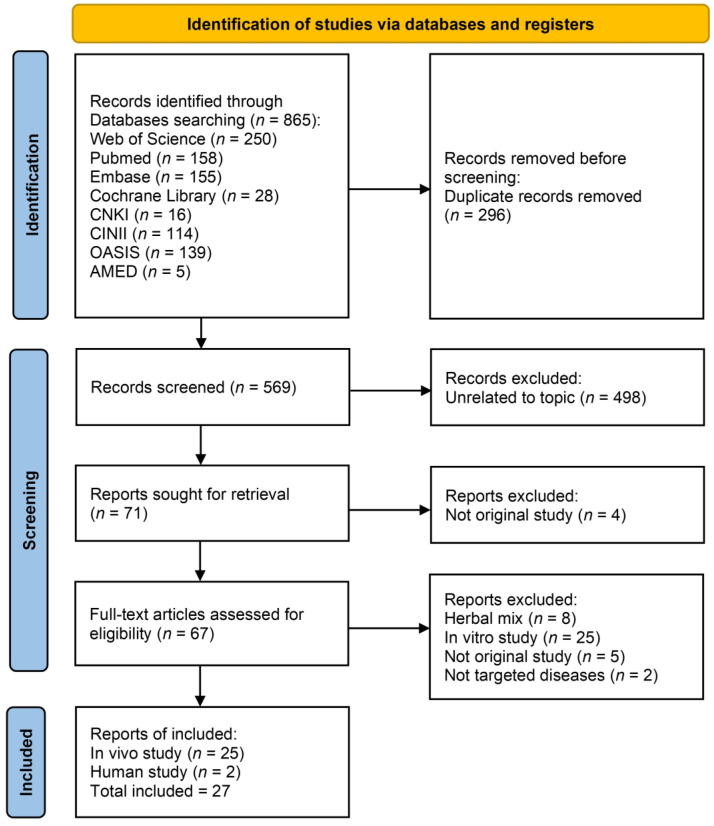
Preferred Reporting Items for Systematic Reviews and Meta-Analyses (PRISMA) flow diagram. Abbreviations: AMED: Allied and Complementary Medicine Database; CiNii: Citation Information by NII; CNKI: China National Knowledge Infrastructure; OASIS: Oriental Medicine Advanced Searching Integrated System.

Ultimately, 27 studies met the eligibility criteria and were retained for final analysis. Of these, 25 were in vivo animal studies and 2 were human studies. The study selection process, including identification, screening, and inclusion, is illustrated in the PRISMA flow diagram (Figure 1).

### 3.2. Characteristics of the Included Studies

#### 3.2.1. Description of the Human Studies

Two clinical trials were incorporated into this review, both investigating the effects of *Dioscoreae Rhizoma* on gastrointestinal health.

Liang et al. [12] investigated the effects of yam porridge intake on intestinal microbiota and immune/clinical indicators in 92 patients undergoing colorectal cancer surgery with type 2 diabetes (T2DM). Patients were randomly assigned to a control group (*n* = 46, standard treatment) or an intervention group (*n* = 46, standard treatment plus yam porridge 150 g/day for 3 weeks).

The integrity of the colonic mucosa during the postoperative recovery phase was evaluated. Serum diamine oxidase (DAO) and D-lactate levels were measured using ELISA-based kits as markers of intestinal barrier damage. Both markers were significantly reduced in the yam group, with DAO at 8.7 ± 2.3 U/L versus 11.2 ± 2.8 U/L and D-lactate at 12.6 ± 3.1 mg/L versus 16.4 ± 3.8 mg/L (both *p* < 0.05), indicating improved mucosal integrity.

Fecal samples were analyzed by 16S rRNA gene sequencing. In the yam group, the relative abundances of *Bifidobacterium* and *Lactobacillus* increased significantly (+23% and +17%, *p* < 0.05), whereas *Enterococcus* and *Escherichia coli* decreased (−14% and −19%, *p* < 0.05). This suggests that yam intake promotes the proliferation of beneficial bacteria while suppressing potential pathogens.

Concentrations of acetate, propionate, and butyrate in fecal samples were quantified by gas chromatography (GC). In the yam group, acetate increased from 41.2 ± 6.7 mmol/kg to 48.9 ± 7.5 mmol/kg, propionate from 18.5 ± 4.3 to 22.7 ± 5.0 mmol/kg, and butyrate from 9.4 ± 2.7 to 12.3 ± 3.1 mmol/kg (all *p* < 0.05), reflecting enhanced intestinal fermentation activity and potential anti-inflammatory effects.

Serum concentrations of Tumor necrosis factor (TNF)-α, Interleukin (IL)-6, and IL-1β were measured by ELISA. All were significantly reduced in the yam group compared with controls: TNF-α (25.3 ± 5.1 vs. 31.7 ± 6.2 pg/mL), IL-6 (18.2 ± 4.5 vs. 23.6 ± 5.4 pg/mL), and IL-1β (14.1 ± 3.7 vs. 17.8 ± 4.1 pg/mL) (*p* < 0.01).

The yam group showed faster wound healing (11.0 ± 1.2 days vs. 13.9 ± 2.2 days, *p* < 0.001) and a lower postoperative infection rate (6.8% vs. 22.2%, *p* = 0.04). Fasting and 2 h postprandial blood glucose levels also improved significantly (*p* < 0.01).

In summary, yam intake improved intestinal barrier function, promoted beneficial microbiota and short-chain fatty acid (SCFA) production, suppressed inflammatory cytokines, and enhanced surgical recovery and metabolic outcomes in patients with T2DM and colorectal cancer.

Pramana et al. [13] conducted a 6-week, open-label, single-arm dietary intervention involving 10 obese adult participants. Subjects consumed a high-fiber functional snack derived from Indonesian *Dioscorea*, designed to deliver antioxidant and fermentable fiber components. The primary endpoint was gut microbiota modulation and fecal samples were analyzed by 16S rRNA gene sequencing. At the end of the study, a notable increase (*p* < 0.05) was detected in beneficial bacterial taxa, including *Bifidobacterium* spp. and *Clostridium coccoides–Eubacterium rectale*, suggesting a prebiotic effect. Changes in body weight, BMI, and blood glucose were minimal and did not reach statistical significance. No adverse events or safety concerns were reported. Although the lack of a control group reduces the interpretability of the findings, the study contributes preliminary human data supporting the role of *Dioscoreae Rhizoma* in promoting favorable shifts in intestinal microbial ecology.

Together, these studies indicate that *Dioscoreae Rhizoma* may support glycemic control, promote gut microbiota balance, and enhance recovery in gastrointestinal conditions. Detailed descriptions of each study’s design, interventions, outcomes, and conclusions are presented in Table 3.

#### 3.2.2. Description of the In Vivo Studies

A total of 25 in vivo studies were included in this review. Most were conducted in rodents: 8 in rats and 14 in mice. The remaining three studies used common carp, rainbow trout, and weaned lamb, respectively. The experimental disease models varied widely, encompassing gastric lesions, colitis, CRC, food allergy, antibiotic-associated diarrhea, and immunosuppression, as well as healthy models aimed at evaluating gastrointestinal and immune functions.

Jeong et al. [14] investigated the gastroprotective effects of *Rhizoma Dioscoreae* extract in ethanol-induced gastric lesion models using Sprague-Dawley (SD) rats. Male SD rats weighing 180–200 g were randomly divided into control and treatment groups (*n* = 8 per group). Ethanol-induced gastric lesions were established by oral administration of 5 mL/kg absolute ethanol. The intervention group received *Rhizoma Dioscoreae* extract orally at a dose of 200 mg/kg/day for 14 consecutive days. Gastric tissues were collected post-treatment, and lesion area was quantified macroscopically. Antioxidant enzyme activity, including Superoxide dismutase (SOD) and Glutathione peroxidase (GPx), was measured using commercial colorimetric assay kits. Over a 14-day oral administration period, the treatment group exhibited a significant increase in antioxidant enzyme activity, including Superoxide dismutase and Glutathione peroxidase, alongside an enhancement in overall antioxidant capacity. These biochemical alterations were associated with a decrease in gastric lesion formation and an increase in stomach weight, indicating that *Dioscorea* extract may mitigate ethanol-induced gastric mucosal damage through antioxidant-mediated pathways.

Park et al. [15] investigated the ulcer-protective potential of *Dioscorea* powder in a cysteamine-induced gastric ulcer model in SD rats. Duodenal ulcers were induced with cysteamine (30 mg/kg, administered twice at a 4 h interval), and *Dioscorea* powder was administered orally. Ulcer index was calculated by measuring lesion length and severity. Protein expression of Cyclooxygenase-2 (COX-2), inducible nitric oxide synthase (iNOS), and CA-IX/XIV was quantified using immunohistochemistry and Western blotting. Following a single oral administration, the treatment resulted in a marked reduction in ulcer index and suppressed inflammatory mediators, including COX-2 and iNOS, while enhancing carbonic anhydrase isozymes IX and XIV. These results highlight *Dioscorea*’s anti-inflammatory and mucosal regulatory effects in chemically induced gastric ulceration.

Byeon et al. [16] utilized *Dioscorea batatas* Decne flesh and peel extracts administered orally to SD rats to evaluate their effect on ethanol-induced acute gastric ulceration. Gastric injury was induced by acute administration of 70% ethanol, after which yam peel or flesh extracts (100–200 mg/kg) were delivered orally. Lesion area was measured macroscopically, mucosal oxidative stress markers such as malondialdehyde (MDA) were quantified, SOD activity was assayed with commercial kits, prostaglandin E2 (PGE2) levels were determined by enzyme immunoassay, and COX-2 expression was examined by Western blotting and immunohistochemistry. Even with a single dose, the treatment group showed increased levels of protective mediators such as SOD, PGE2, and COX-2, accompanied by reduced oxidative stress and smaller gastric lesions. This supports the notion that *Dioscorea*-based compounds may provide immediate mucosal protection by boosting endogenous antioxidant and prostaglandin pathways.

Mao et al. [17] explored the efficacy of the extract of Winged yam Resistant Starch Type 2 in ethanol-induced gastric ulceration using Kunming mice. The Winged yam Resistant Starch Type 2 fraction was administered orally for seven days prior to ethanol exposure. Ulcer index was calculated by measuring lesion length, oxidative stress markers (MDA and SOD) were assessed using colorimetric assays, and gastric mucosa was evaluated histologically for protective changes. After a 7-day oral treatment regimen, significant reductions in ulcer index and MDA levels were observed, alongside elevated SOD activity. These findings suggest that *Dioscorea* supplementation may prevent gastric mucosal injury via oxidative stress attenuation and tissue preservation.

Guo et al. [18] investigated the protective potential of a Chinese *Dioscorea*-derived preparation (Chinese iron yam polysaccharide, CIYP) in a BALB/c mouse model of ethanol-induced gastric mucosal lesion. CIYP was purified through hot water extraction, deproteinization, anion-exchange chromatography, and gel filtration, yielding a triple-helix polysaccharide (average Mw ≈ 1.67 × 10^3^ kDa). Mice were pre-treated orally with CIYP at 200 or 400 mg/kg/day for 14 days before ethanol exposure. Gastric lesion index and inhibition rate were calculated, while mucosal NO, PGE2, and epidermal growth factor (EGF) were quantified. Antioxidant capacity was assessed via SOD and MDA assays, and mucosal morphology was evaluated using H&E and PAS staining. Structural characterization of CIYP was further confirmed with scanning electron microscopy, atomic force microscopy, fourier transform infrared, and nuclear magnetic resonance spectroscopy. The treatment group showed significant suppression of gastric damage, as evidenced by reduced mucosal inflammation. Mechanistically, the study reported increased mitogen-activated protein kinase signaling and decreased expression of inflammatory mediators. These findings suggest that CIYP may protect against ethanol-induced gastric injury by modulating inflammatory pathways.

Xie et al. [18] explored the protective potential of compound yam water extract (CYW), a *Dioscorea*-derived compound, in ICR mice with ethanol-induced gastric injury. Acute injury was induced with oral ethanol (10 mL/kg), and CYW was administered either as pre-treatment or post-treatment to compare preventive versus therapeutic effects. Serum and tissue cytokine levels were determined by ELISA, antioxidant enzyme activity was measured with biochemical kits, and Bcl-2/Bax, vascular endothelial growth factor (VEGF), and TGF-β1 expression was analyzed via qPCR and Western blotting. Mucosal lesions were further evaluated using H&E and mucus staining. Following a 14-day oral administration of 10 mL/kg ethanol, the treatment group exhibited notable reductions in inflammatory cytokines (TNF-α, IL-6, and IL-1β) and oxidative stress, alongside elevations in antioxidant enzymes such as SOD, catalase (CAT), and GPx. Histological evaluation showed improved mucosal architecture, and molecular analysis revealed upregulation of Bcl-2/Bax, Vascular Endothelial Growth Factor, and Transforming Growth Factor-β1. These results indicate that CYW exerts gastroprotective activity mediated by anti-inflammatory, antioxidant, and tissue-repair mechanisms.

Jeon, J.R. et al. [19] assessed the impact of Chinese *Dioscorea* ethanol extract on gastrointestinal motility and metabolic indices in SD rats under normal physiological conditions. Male SD rats (weighing 180–200 g) were divided into groups receiving either distilled water (control) or Chinese yam ethanol extract (100 or 200 mg/kg/day, orally) for six weeks. Gastrointestinal transit was measured by charcoal meal passage, gastric acid secretion was quantified by pylorus ligation method, fecal output was collected daily, and intestinal microflora composition was analyzed by selective culture. Blood glucose and lipid profiles were determined by enzymatic assays. Histological analysis of gastric and colonic mucosa was also conducted to evaluate tissue-level changes. After 6 weeks of oral administration, the extract significantly improved gastrointestinal transit, fecal output, and glycemic control. Mechanistic insights included reduced gastric acid secretion, increased colonic motility, enhanced proliferation of lactose-fermenting bacteria, and decreased systemic glucose and lipid levels. These findings highlight the potential role of *Dioscorea* in improving gut function and metabolic regulation under non-pathological conditions.

Jeon, J.R. et al. [20] investigated the laxative effects of *Dioscorea* yogurt in SD rats with constipation induced by loperamide. Constipation was induced by oral loperamide (3 mg/kg/day) for three days, after which rats were treated with yogurt containing *Dioscorea* powder (5 g/kg/day) for five days. Fecal parameters (moisture, weight, and frequency) were measured daily, histological analysis of colonic tissue was performed using Alcian Blue staining for goblet cells, and Ki-67 expression was assessed by immunohistochemistry. Mucin levels were quantified biochemically, confirming mucosal secretion enhancement. The intervention significantly increased goblet cell count, mucin production, and expression of Ki-67, a marker of cellular proliferation. These effects translated into improved fecal moisture content and evacuation frequency. The study suggests that *Dioscorea*-based functional food may alleviate functional constipation through mucus secretion and epithelial turnover enhancement.

Nishimura et al. [21] explored the lipid-lowering effects of red and black *Dioscorea* varieties in SD rats under normal physiological conditions. Male SD rats were fed a basal diet supplemented with 5% red or black yam powder for three weeks. Serum lipid profiles were analyzed enzymatically, hepatic MTP mRNA expression was quantified via RT-PCR, and SCFA concentrations in cecal contents were determined by gas chromatography. Fecal lipid content and total bile acid excretion were also measured to evaluate lipid metabolism. Over a 3-week dietary intervention period, *Dioscorea* supplementation led to a significant reduction in non-high-density lipoprotein (HDL) cholesterol and hepatic microsomal triglyceride transfer protein mRNA expression. Concurrently, SCFA production was elevated, indicating enhanced colonic fermentation. These findings suggest that *Dioscorea* intake may support lipid metabolism and cholesterol regulation even in the absence of pathological stimuli.

Wang et al. [22] investigated the prebiotic potential of raw *Dioscorea* in BALB/c mice over a 21-day dietary administration. Mice were fed chow supplemented with 10% raw yam powder for three weeks. Fecal parameters were measured daily, SCFA concentrations in cecal contents were analyzed by HPLC, and gut microbiota composition was assessed by selective plating for *bifidobacteria* and *lactobacilli*. Colonic morphology was examined histologically (crypt depth and goblet cell count), and epithelial barrier integrity was further evaluated by tight junction protein immunohistochemistry. *Dioscorea* consumption led to increased fecal moisture, elevated SCFA levels, and enhanced crypt depth in the colonic mucosa. Moreover, the study observed significant enrichment of beneficial microbial taxa such as *bifidobacteria* and improved intestinal barrier function. These results demonstrate that raw *Dioscorea* may beneficially modulate the gut microenvironment and epithelial structure, exerting a prebiotic effect.

Hsu et al. [23] investigated the effects of dietary Chinese/Japanese *Dioscorea* in a BALB/c mouse model of lipopolysaccharide-induced intestinal damage. Methodologically, mice received either a control diet or diets supplemented with 5% Chinese yam or Japanese yam powder for 4–8 weeks before receiving intraperitoneal lipopolysaccharide (LPS) (5 mg/kg). Enzyme assays (SOD and sucrase), lipid peroxidation (MDA), and microbial counts were determined from cecal contents. Intestinal histology was assessed for mucosal damage and repair. Animals fed *Dioscorea* for 4–8 weeks exhibited elevated antioxidant enzyme activity (e.g., SOD), reduced MDA levels, increased sucrase activity, and a significant reduction in *Clostridium perfringens* populations. These findings suggest that *Dioscorea* exerts protective effects through both antioxidant mechanisms and modulation of gut microbiota, highlighting its potential role in maintaining intestinal health.

Chen et al. [24] examined the antioxidant and anticancer effects of *Dioscorea* bulbifera (DB) ethanol extract in C57BL/6 mice with 2,4,6-Trinitrobenzenesulfonic acid (TNBS)-induced colitis. Here, colitis was induced by intra-rectal instillation of TNBS (2.5 mg in 50% ethanol), followed by daily intra-rectal DB extract administration (50–100 mg/kg) for 7 days. Oxidative stress markers (MDA and SOD) and histology were assessed, while cancer-related protein expression (including SGC-7901 markers) was analyzed via immunohistochemistry and Western blot. The treatment, administered via an intra-rectal route, resulted in a reduction in oxidative stress markers and suppressed cancer cell proliferation, notably reducing the expression of SGC-7901, a gastric carcinoma cell line. These outcomes indicate that DB extract may possess dual antioxidant and anticancer activities relevant to colonic inflammation and tumorigenesis.

Chen et al. [25] examined the anti-inflammatory properties of *Dioscorea alata* L. anthocyanins (DACNs), a *Dioscorea*-derived compound, in a TNBS-induced colitis model using C57BL/6 mice. DACNs (100 mg/kg) were administered intra-rectally during TNBS (2.5 mg) induction in C57BL/6 mice. Cytokines (TNF-α, Interferon [IFN]-γ, and IL-6) and iNOS were quantified by ELISA and RT-PCR, tight junction proteins (zonula occludens-1 [ZO-1] and occludin) were analyzed immunohistochemically, and histological scoring was performed to evaluate mucosal integrity. When administered intra-rectally during TNBS induction, DACNs significantly downregulated pro-inflammatory cytokines, including TNF-α, IFN-γ, and iNOS, while enhancing intestinal tight junction protein expression. Improved histological scores suggest that DACNs confer mucosal protection and exert anti-inflammatory effects in colitis through both immunomodulatory and barrier-enhancing mechanisms.

Cai et al. [26] investigated the colitis-modulating effect of CP, a combination of *Dioscorea* polysaccharide and inulin, in SD rats with TNBS-induced colitis. Colitis was induced with intra-rectal TNBS (2.5 mg/rat), and CP was administered orally (200 mg/kg/day) for 16 days. Disease activity index (DAI) scores were recorded daily, MPO activity was assayed spectrophotometrically, SCFAs were measured by gas chromatography, and colonic mucosa was examined histologically with H&E and Alcian Blue. Following 16 days of oral administration, the treatment group exhibited reduced disease activity index, lower myeloperoxidase activity, and enhanced colonic histology. SCFA levels and lactic acid bacteria populations were also elevated, suggesting that CP may alleviate intestinal inflammation and improve colonic integrity via fermentation-mediated microbiota enhancement.

Chung-Hsiung et al. [27] explored the immunomodulatory effects of diosgenin, a bioactive *Dioscorea* component, in a BALB/c mouse model of ovalbumin (OVA)-induced food allergy. Mice were administered yam polysaccharides orally at doses of 50 or 100 mg/kg/day for 4 weeks. Splenic lymphocyte proliferation was assessed using a 3H-thymidine incorporation assay after stimulation with ConA and LPS, macrophage phagocytosis was quantified by uptake of fluorescent latex beads, and NK cell activity was measured using a chromium-release assay against YAC-1 target cells. Serum cytokines (IL-2 and IFN-γ) were measured by ELISA, and spleen histology was performed to assess structural changes. Over 13 days of oral administration, diosgenin supplementation led to an upregulation of regulatory T cell (Treg)-associated markers including IL-10, Forkhead box P3, IFN-γ, and Type 1 T helper (Th1) chemokines. This was accompanied by a significant suppression of allergic inflammation, suggesting that diosgenin may exert anti-allergic effects by promoting Th1/Treg immune responses.

Zhang et al. [28] assessed the gut-restorative effects of *Dioscorea* suspension in BALB/c mice with antibiotic-associated diarrhea (AAD) induced by ampicillin. Male BALB/c mice (6–8 weeks old, 18–22 g) were randomized into control and treatment groups (*n* = 10 each). AAD was induced by intragastric administration of ampicillin (1 g/kg/day) for 5 consecutive days. Following induction, the treatment group received oral gavage of *Dioscorea* suspension (400 mg/kg/day) for 10 days. Fecal samples were collected daily, and SCFAs were quantified using GC. Gut microbiota composition was assessed by 16S rRNA sequencing. Clinical diarrhea severity was scored based on stool consistency and frequency. After 10 days of oral treatment, mice receiving *Dioscorea* exhibited increased levels of SCFAs and enhanced diversity of gut microbiota, including beneficial bacteria. Clinically, diarrhea symptoms were markedly alleviated. These findings support the potential of *Dioscorea*-based interventions in recovering intestinal microbiota balance and relieving antibiotic-related gastrointestinal side effects.

Meng et al. [29] assessed the effects of *Dioscorea* peel supplementation in a healthy feeding trial using common carp. Common carp with an average initial weight of 15 ± 2 g (*n* = 60 per group) were randomly assigned into control and treatment groups. Fish in the treatment group received a basal diet supplemented with 5% *Dioscorea* peel powder, while the control group was fed only the basal diet. The feeding trial lasted for 8 weeks, during which fish were maintained in recirculating aquaculture tanks at 25 ± 1 °C with continuous aeration. At the end of the intervention, intestinal samples were collected for histological examination, and tight junction proteins (ZO-1 and occludin) were analyzed by Western blotting. Antioxidant enzyme activity (SOD, CAT, and lysozyme) was measured using spectrophotometric kits, and SCFA concentrations were determined by HPLC. Microbiota composition was assessed by 16S rRNA sequencing to quantify changes in *Lactobacillus* and *Enterobacteriaceae* abundance. Over an 8-week period, *Dioscorea* peel intake led to significant increases in antioxidant enzyme activity (SOD, CAT, and lysozyme), expression of tight junction proteins (ZO-1 and occludin), and levels of SCFAs and *Lactobacillus*, while decreasing *Enterobacteriaceae* and pro-inflammatory markers including nuclear factor kappa-light-chain-enhancer of activated B cells (NF-κB) and IL-1β. These results indicate that *Dioscorea* peel may enhance intestinal barrier integrity and immune competence in aquatic species through microbiota modulation and antioxidant mechanisms.

Wang et al. [30] investigated the immunostimulatory effects of *Dioscorea* extract in rainbow trout under healthy conditions. Juvenile rainbow trout (Oncorhynchus mykiss, average body weight 12 g, *n* = 40 per group) were reared in tanks and assigned to diets supplemented with *Dioscorea* extract (2% *w*/*w*) or basal diet for 56 days. At the end of feeding, serum samples were collected for immunological assays. Cytokine levels (IL-2, IL-6, and TNF-α) were determined using ELISA, and antioxidative enzyme activities (GPx1 and CAT) were assessed spectrophotometrically. Heat shock protein expression was determined by Western blot, and intestinal microbiota was analyzed via culture-dependent methods and 16S rRNA sequencing. Over a 56-day feeding period, *Dioscorea* extract promoted increases in digestive and immune-related enzymes (e.g., IL-2, IL-6, TNF-α, complement C4), heat shock proteins, and antioxidative enzymes such as GPx1. Additionally, levels of beneficial gut microbes such as *Lactobacillus* and *Bifidobacterium* were elevated. These findings suggest that *Dioscorea* extract contributes to enhanced gut immunity and microbial balance, supporting its role in immunonutrition for aquatic health.

Huang et al. [31] evaluated the effects of SCYP, a *Dioscorea*-based preparation, on cyclophosphamide (CTX)-induced immunosuppression in BALB/c mice. Mice (*n* = 10 per group) were injected intraperitoneally with CTX (80 mg/kg) once daily for 3 days to induce immunosuppression. The treatment group then received oral SCYP supplementation (300 mg/kg/day) for 7 days. Fecal samples were analyzed for microbial composition by 16S rRNA sequencing, while enzymatic activities of intestinal amylase, protease, and lipase were measured spectrophotometrically. Serum samples were collected to evaluate immune indices. Following 7 days of oral administration, SCYP significantly enhanced gut microbial diversity, marked by increased levels of *Lactobacillus* and *Akkermansia*, along with a reduction in *Proteobacteria*. Digestive enzyme activity also improved. These outcomes indicate that *Dioscorea* supplementation can restore gut ecological balance and support mucosal enzymatic function in states of immunosuppression.

Kweon et al. [32] examined the anti-inflammatory potential of *Dioscorea*-derived DB extract in a caerulein-induced pancreatitis model using C57BL/6 mice. Male mice (20–22 g, *n* = 8 per group) were injected intraperitoneally with caerulein (50 μg/kg) every hour for 6 h to induce acute pancreatitis. The treatment group was administered *Dioscorea* extract (250 mg/kg, oral gavage) 1 h prior to the first caerulein injection. Serum samples were analyzed for pancreatic enzyme (lipase and amylase) activity, and inflammatory cytokines (IL-6, TNF-α, and IL-1β) were quantified using ELISA. Pancreatic tissues were collected for histopathological scoring. Within just 6 h of oral administration, the treatment group showed decreased pancreatic enzyme (lipase) activity and reduced expression of inflammatory cytokines (IL-6 and TNF-α), while IL-1β levels remained unchanged. A reduction in pancreas index and histopathological damage confirmed the therapeutic potential of *Dioscorea* extract in ameliorating acute pancreatic inflammation.

Lu et al. [33] investigated the immunomodulatory effects of *Dioscorea* polysaccharide in ICR mice subjected to CTX-induced immunosuppression. ICR mice (*n* = 10 per group) were injected intraperitoneally with CTX (100 mg/kg) for 3 consecutive days, followed by oral *Dioscorea* polysaccharide supplementation (400 mg/kg/day) for 28 days. Thymus and spleen indices were calculated by organ weight relative to body weight. Flow cytometry was used to analyze CD4^+^/CD8^+^ T cell ratios. Immunoglobulin (IgA, IgG, and IgM) levels were determined by ELISA, while colonic tissues were examined for tight junction protein expression (Western blot for ZO-1 and occludin). SCFA concentrations were analyzed using GC-MS, and tryptophan metabolites were quantified by LC-MS. Over a 28-day oral treatment, mice exhibited increases in thymus index, CD4^+^/CD8^+^ T cell ratios, immunoglobulins (IgA, IgG, IgM), tight junction proteins (ZO-1 and occludin), SCFAs, and tryptophan metabolism. These results suggest that *Dioscorea*-derived polysaccharides can effectively restore immune function and intestinal integrity under pharmacologically induced immune suppression.

Kinoshita et al. [34] evaluated the antitumor potential of dietary *Dioscorea* powder in a BALB/c mouse model of 1,2-dimethylhydrazine (DMH)-induced aberrant crypt foci (ACF), a precursor lesion to colon cancer. Mice (*n* = 12 per group) received intraperitoneal injections of DMH (20 mg/kg) once weekly for 6 weeks to induce ACF, while the treatment group was fed a diet supplemented with 5% *Dioscorea* powder for 8 weeks. Colonic tissues were collected, and ACF number was quantified microscopically. Apoptosis-related genes (Bax, Bcl-2, and caspase-3) were analyzed by RT-PCR. Over 8 weeks of diet-based intervention, *Dioscorea* consumption led to significant downregulation of ACF formation and upregulation of apoptosis-related genes in colonic tissue. This suggests that *Dioscorea* intake may exert chemo-preventive effects against colorectal tumorigenesis through apoptosis-mediated pathways.

Kinoshita et al. [31] explored the chemopreventive potential of a dietary preparation consisting of Chinese yam and leaf juice (*Aojiru*) in BALB/c mice subjected to 1,2-DMH-induced ACF. Mice (*n* = 12 per group) received intraperitoneal injections of DMH (20 mg/kg) once weekly for 6 weeks to induce ACF, while the treatment group was fed a diet supplemented with 5% *Dioscorea* powder for 8 weeks. Colonic tissues were collected, and ACF number was quantified microscopically. Apoptosis-related genes (Bax, Bcl-2, and caspase-3) were analyzed by RT-PCR. Over a 4-week dietary intervention, the treatment group demonstrated a marked reduction in ACF formation, alongside a significant increase in apoptotic activity within the colonic epithelium. These findings suggest that yam-based dietary supplementation may exert protective effects against early neoplastic lesions in the colon via apoptosis enhancement mechanisms.

Son et al. [35] assessed the anti-carcinogenic properties of a *Dioscorea*-based diet in an azoxymethane (AOM)-induced colon cancer model using F344 rats. Male rats (5 weeks old, *n* = 8 per group) were administered AOM (15 mg/kg, intraperitoneally, once weekly for 2 weeks). The treatment group was fed a basal diet containing 10% *Dioscorea* powder for 10 weeks. Colonic tissues were collected for histopathological scoring, and ACF counts were determined microscopically. Antioxidant enzyme activities (glutathione [GSH], GPx, CAT, and Cu/Zn-SOD) were measured spectrophotometrically. Expression of inflammatory mediators (COX-2, NF-κB, IL-1β, and TNF-α) was assessed by Western blot. After 10 weeks of oral intervention, the treatment group exhibited a significant reduction in ACF formation and colonic tumor markers. Biochemical analysis revealed increased antioxidant enzyme activity (GSH, GPx, CAT, and Cu/Zn-SOD) and suppression of pro-inflammatory and tumorigenic mediators including COX-2, NF-κB, IL-1β, and TNF-α. These data collectively indicate that *Dioscorea* intake may attenuate AOM-induced colorectal carcinogenesis through antioxidant and anti-inflammatory mechanisms.

Yang et al. [36] proved that *Dioscorea* opposite waste (DOW) improves the gastrointestinal microbiome, and shows antioxidation capacity and immune activity. Sixty healthy male small tail Han lambs were equally assigned to four dietary treatments: DOW-free diet, and addition of 10%, 15% and 20% DOW diet. Experimental lambs were fed a corresponding diet for 62 days and rumen microbiome and plasma parameters were determined at the end of the experiment. The results showed that DOW linearly increased the concentration of aspartate aminotransferase (AST), alkaline phosphatase (ALP), IgA, IgM, IgG, GPx, SOD and total antioxidant capacity, but decreased IL-1β, IL-6, TNF-α, and MDA. Sequencing of rumen metagenome revealed that DOW increased relative abundance of phylum *Verrucomicrobia*, *Planctomycetes*, *Fibrobacteres*, *Chloroflexi*, *Actinobacteria*, and *Acidobacteria* and species *Ruminococcaceae_*bacterium, *Clostridiales_*bacterium_NK3B98, *Clostridiales_*bacterium, and *Clostridia_*bacterium. DOW increased the immune response and antioxidant capacity in a dose-dependent manner and modulated the composition of rumen microbiome function with improving the polysaccharide hydrolase activity in the rumen.

Notably, none of the included studies reported any significant adverse events, suggesting a favorable safety profile for *Dioscorea* interventions. A summary of each study’s treatment, mechanisms, outcomes, and efficacy is presented in Table 4.

### 3.3. Risk of Bias Assessment for Included Human Studies

The risk of bias assessment for the two human studies included in this review was conducted using two different tools. For the randomized trial by Liang et al. [12], the Cochrane RoB 2 was applied. For the non-randomized study by Pramana et al. [13], the ROBINS-I V2 tool was used.

#### 3.3.1. Bias Arising from the Randomization Process

Liang et al. [12] reported the use of IBM SPSS 25.0 software for random allocation but did not specify the method used to generate the random sequence or whether any restrictions (e.g., block or stratified randomization) were applied. Due to insufficient methodological detail, this study was judged as having some concerns regarding the risk of bias in this domain. In the case of Pramana et al. [13], randomization was not employed, as the study followed a non-randomized before–after comparison design. Therefore, this domain is not applicable (N/A) under the ROBINS-I framework.

#### 3.3.2. Bias Due to Deviations from Intended Interventions

Liang et al. [12] employed an open-label design in which participants were aware they were receiving yam gruel as part of their dietary intervention. No blinding of participants or personnel was implemented. Consequently, the risk of deviations from the intended intervention was considered substantial, and the study was rated as having a high risk of bias in this domain. For Pramana et al. [13], no information was provided regarding monitoring or control of deviations from the intervention (i.e., snack consumption), and there was no mention of adherence checks or protocol compliance. As a result, this domain was judged as having a serious risk of bias in the ROBINS-I assessment due to potential unmeasured deviations.

#### 3.3.3. Bias Due to Missing Outcome Data

In Liang et al. [12], three participants (two in the intervention group and one in the control group) were lost to follow-up, with reasons for dropout clearly documented. Given the low attrition and transparent reporting, the study was assessed as having a low risk of bias in this domain. In contrast, Pramana et al. [13] initially recruited 69 participants but analyzed data from only 10 (five per group). The reasons for excluding the majority were not adequately explained, and the high exclusion rate raises concerns about bias due to missing data. Therefore, this domain was assessed as having a serious risk of bias under ROBINS-I.

#### 3.3.4. Bias in Measurement of the Outcome

Liang et al. [12] assessed primary clinical outcomes (e.g., wound healing) using clinicians reportedly blinded to group assignment, and laboratory outcomes (e.g., blood glucose) were measured via objective methods. Thus, the study was judged to have a low risk of bias in this domain. Pramana et al. [13] utilized real-time quantitative polymerase chain reaction to assess gut microbiota composition—an objective outcome. However, there was no mention of blinding of outcome assessors, and the small sample size may have compromised result stability. Consequently, this domain was rated as having a moderate risk of bias under ROBINS-I.

#### 3.3.5. Bias in Selection of the Reported Result

Neither study had a pre-registered protocol or publicly available statistical analysis plan. Although both reported the outcomes described in their respective methods sections, the lack of a predefined plan raises the potential for selective reporting. Therefore, Liang et al. rated it as having some concerns [12], and Pramana et al. assessed it as having a serious risk of bias [13] in this domain.

#### 3.3.6. Bias Due to Confounding

This domain is not applicable under the Cochrane RoB 2 framework. The study by Pramana et al. [13] used a non-randomized before–after design without statistical adjustments for potential confounders such as dietary intake, physical activity, comorbidities, or medication use. Although no significant baseline differences were reported, notable variation in age, weight, and BMI was observed across groups. Given the small sample size (*n* = 5 per group) and the absence of covariate adjustment, confounding effects could not be ruled out. This domain was therefore rated as having a serious risk of bias under ROBINS-I.

#### 3.3.7. Bias Due to Selection of Participants

This domain is not applicable under the Cochrane RoB 2 framework. Pramana et al. [13] initially recruited 69 participants, but only 10 (five per group) were included in the final analysis after post hoc exclusions based on self-reported antibiotic or probiotic use. The selection procedure lacked a prespecified, consistently applied protocol, raising concerns about selection bias before intervention exposure. The final sample may not reflect the broader target population. Therefore, this domain was judged to have a serious risk of bias.

#### 3.3.8. Bias in Classification of Interventions

Although Pramana et al. [13] clearly differentiated between the standard and test snacks, the study lacked details regarding the composition, preparation, and administration of the interventions. There was no evidence of procedures to verify adherence or ensure consistent delivery. Given the open-label design, both participants and investigators were likely aware of group assignments, increasing the risk of misclassification or performance bias. Thus, this domain was judged to have a serious risk of bias.

#### 3.3.9. Overall Risk of Bias Assessment

**Figure 2:**
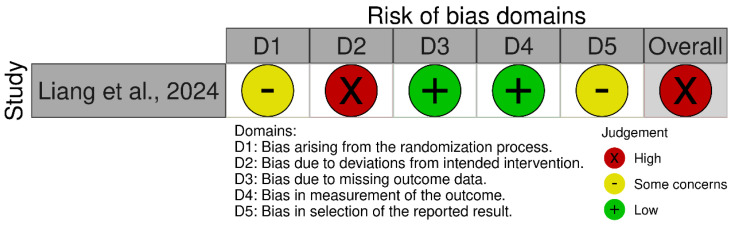
Risk of bias assessment for the randomized human study using the Cochrane Risk of Bias Tool 2.0 [12].

**Figure 3:**
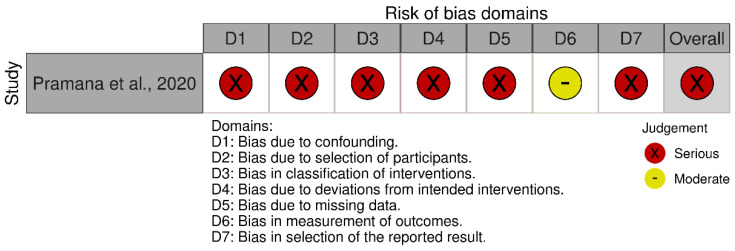
Risk of bias assessment for the non-randomized human study using the ROBINS-I Version 2 tool [13].

For Liang et al. [12], despite low risk assessments in several domains, the presence of high risk of bias due to deviations from the intended interventions led to an overall judgment of high risk of bias, in accordance with the Cochrane RoB 2.0 criteria. For Pramana et al. [13], the ROBINS-I V2 assessment determined an overall serious risk of bias, based on multiple domains showing methodological limitations. Specifically, the study did not adjust for potential confounding variables (D1), and the participant selection process lacked clarity, raising concerns about selection bias (D2). There was insufficient reporting on the classification and delivery of interventions (D3), and no adherence checks or monitoring of protocol compliance were described (D4). Missing outcome data were not adequately addressed (D5), and the absence of a prespecified analysis plan increased the likelihood of selective outcome reporting (D7). Although the measurement of outcomes (D6) was judged to have a moderate risk of bias, the cumulative effect of serious risks in the other domains led to an overall assessment of serious risk of bias. The summary of the risk of bias assessment for each study is depicted in Figure 2 [12] and Figure 3 [13].

### 3.4. Risk of Bias Assessment for Included Animal Studies

The methodological quality of the included animal studies (*n* = 25) was assessed using the SYRCLE’s RoB tool. Results are summarized in Supplementary Table S1.

#### 3.4.1. Sequence Generation

Of the 25 studies, 8 clearly described appropriate random sequence generation (e.g., random number tables, lottery methods), and were rated as low risk of bias. Twelve studies used vague terms like “randomization” without methodological clarification and were rated as having some concerns. Four studies provided no information on sequence generation and were classified as no information. One study explicitly reported non-random or intentional assignment methods and were rated as high risk of bias.

#### 3.4.2. Baseline Characteristics

Among the 25 studies, 21 reported no significant baseline differences or demonstrated adequate balance between groups, and were thus rated as low risk of bias. Four studies lacked statistical comparison or used vague descriptions, resulting in a rating of “some concerns.” None of the studies were categorized as providing “no information” or high risk of bias.

#### 3.4.3. Allocation Concealment

Only 5 studies described allocation concealment procedures and were rated as low risk of bias. Three studies hinted at concealment but lacked detail, resulting in a rating of “some concerns.” The remaining 17 studies made no mention of allocation concealment and were rated as “no information.”

#### 3.4.4. Random Housing

Sixteen studies provided no information on housing practices (e.g., random or treatment-group housing), and were rated as no “information.” Four studies offered unclear statements such as “housed separately,” leading to a rating of “some concerns.” The remaining five studies explicitly reported random housing or cage allocation, and were rated as low risk of bias.

#### 3.4.5. Blinding of Caregivers and Investigators

Only four studies described appropriate blinding procedures for caregivers or investigators and were judged to have a low risk of bias. Four studies used terms such as “blinded” or “double-blind” without adequate elaboration and were categorized as having “some concerns.” Sixteen studies lacked any information on this aspect and were marked as “no information.” Additionally, one study explicitly indicated that outcomes were assessed without blinding or involved highly subjective endpoints and was therefore considered at high risk of bias.

#### 3.4.6. Random Outcome Assessment

Four studies clearly reported randomized outcome assessment and were classified as having a low risk of bias. Fourteen studies did not provide sufficient information regarding the order of measurements or assessor independence and were rated as “no information.” The remaining seven studies were judged to have “some concerns” due to vague descriptions or insufficient procedural detail.

#### 3.4.7. Blinding of Outcome Assessment

Two studies reported assessor blinding or employed objective measurements and were rated as having a low risk of bias. Sixteen studies were judged to have “some concerns” due to limited information on the blinding of outcome assessors. Five studies lacked any information on this domain and were categorized as “no information.” Additionally, two studies explicitly stated that outcomes were assessed without blinding or used highly subjective endpoints and were therefore considered to be at high risk of bias.

#### 3.4.8. Incomplete Outcome Data

Among the 25 included animal studies, 23 either reported no attrition or provided clear and adequate justifications for excluded data and were therefore assessed as having a low risk of attrition bias. In contrast, two studies lacked sufficient explanation for missing or incomplete outcome data and were thus rated as having a high risk of bias in this domain.

#### 3.4.9. Selective Outcome Reporting

Fourteen studies reported prespecified outcomes or showed consistency with the study objectives and were rated as having a low risk of bias. Nine studies did not provide sufficient detail to evaluate the completeness of reporting and were judged as having “some concerns.” Two studies omitted critical endpoints without justification and were considered at high risk of reporting bias.

#### 3.4.10. Other Sources of Bias

Sixteen studies fulfilled at least two quality assurance criteria, such as sample size justification, ethical approval, or appropriate statistical analysis, and were therefore considered to have a low risk of bias. Seven studies raised “some concerns” due to incomplete methodological transparency or potential conflicts of interest. Two studies lacked sufficient information on these aspects and were marked as “no information.”

## 4. Discussion

### 4.1. Summary of Key Findings

**Figure 4:**
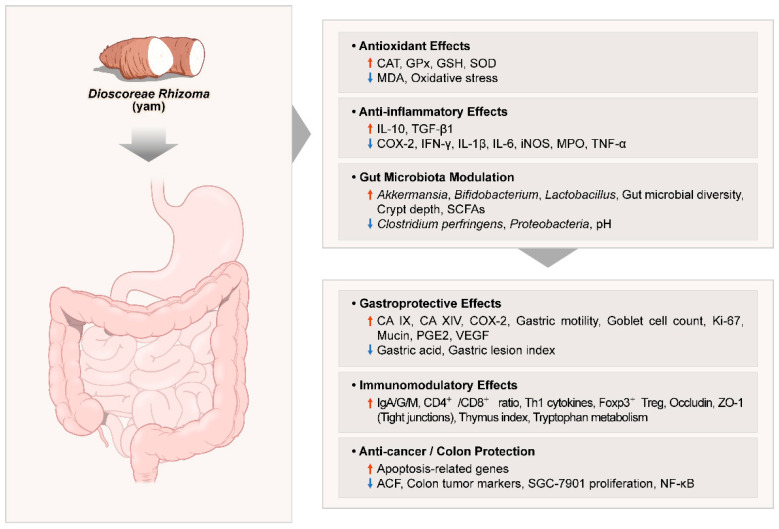
Summary of the molecular mechanisms underlying the gastrointestinal and metabolic effects of *Dioscoreae Rhizoma*. Abbreviations: ↑: significantly increased, ↓: significantly decreased; ACF: aberrant crypt foci; CA IX/XIV: carbonic anhydrase isoforms IX and XIV; CAT: catalase; COX-2: cyclooxygenase-2; GPx: glutathione peroxidase; GSH: glutathione; IFN: interferon; Ig: immunoglobulin; IL: interleukin; iNOS: inducible nitric oxide synthase; MDA: malondialdehyde; MPO: myeloperoxidase; NF-κB: nuclear factor kappa-light-chain-enhancer of activated B cells; PGE2: prostaglandin E2; SCFA: short-chain fatty acids; SOD: superoxide dismutase; TGF: transforming growth factor; Th1: Type 1 T helper; TNF: tumor necrosis factor; Treg: regulatory T cell; VEGF: vascular endothelial growth factor; ZO-1: zonula occludens-1.

This study reviewed human trials and synthesized a wide range of in vivo studies investigating the therapeutic potential of *Dioscoreae Rhizoma* in gastrointestinal health. Key findings include gastroprotective effects against ethanol-induced gastric lesions, improvements in gastrointestinal function and motility, and enhanced SCFA production through modulation of gut microbiota and increased populations of beneficial bacteria. Anti-colitic and anti-inflammatory effects were consistently observed in models of colitis, antibiotic-associated diarrhea, pancreatitis, and immune dysfunction, highlighting the immunomodulatory capacity of *Dioscoreae Rhizoma*. Additionally, chemo-preventive properties were observed through the reduction in colon tumor markers and modulation of apoptosis and inflammatory pathways. Collectively, these studies underscore the multifaceted bioactivity of *Dioscoreae Rhizoma*, particularly via antioxidant and anti-inflammatory mechanisms, enhancement of the gut barrier, and immune regulation, supporting its clinical relevance in gastrointestinal disorders. A visual summary of the overall mechanisms of *Dioscoreae Rhizoma* is presented in Figure 4, highlighting the integrated antioxidative, anti-inflammatory, and gut microbiota modulatory pathways.

### 4.2. Predominant Mechanisms of Action

#### 4.2.1. Mechanisms of Gastroprotection and Mucosal Barrier Enhancement

One of the most frequently reported mechanisms involves the antioxidant and anti-inflammatory properties of *Dioscorea*. For example, studies [14,17,18] showed that *Dioscorea* administration in ethanol-induced gastric ulcer models significantly increased the activity of antioxidant enzymes such as SOD, GPx, and CAT, while reducing pro-inflammatory cytokines like TNF-α, IL-1β, and IL-6. These biochemical changes contributed to the protection and repair of the gastric mucosa.

Another key mechanism involves the reinforcement and regeneration of the intestinal mucosal barrier. Studies [15,16,20,37] demonstrated that *Dioscorea* enhanced goblet cell counts, mucin secretion, and Ki-67 expression, along with the upregulation of tight junction proteins such as zonula occludens-1 and occludin. These findings suggest that *Dioscorea* plays a role in maintaining mucosal integrity and regulating intestinal permeability.

Through these mechanisms, *Dioscorea* has shown therapeutic potential in various gastrointestinal disease models by reducing inflammation, inhibiting gastric acid secretion, protecting the gastric lining, and modulating intestinal motility [14,15,16,17].

#### 4.2.2. Effects on Gut Microbiota and Colonic Environment

The influence of *Dioscorea* on gut microbiota was highlighted in a clinical study [13], where consumption of a high-fiber *Dioscorea*-based snack led to a significant increase in beneficial bacteria such as *Bifidobacterium* spp. and *Clostridium coccoides–Eubacterium rectale*, indicating a potential prebiotic effect and its role in enhancing microbial diversity.

Additionally, studies [21,22,29] reported increased SCFA production, enhanced colonic crypt depth, enrichment of beneficial microbiota, and suppression of abnormal metabolic byproducts. These outcomes support the potential of *Dioscorea* in managing functional constipation and irritable bowel syndrome.

#### 4.2.3. Comprehensive Categorization by Experimental Models

This review categorizes and evaluates the effects of *Dioscorea* based on experimental models, including gastritis (ethanol-induced lesions), ulcers (cysteamine-induced), colitis (TNBS-induced), constipation (loperamide-induced), gut microbiota modulation (AAD and healthy models), and immune regulation (CTX and OVA-induced).

### 4.3. Comparison with Existing Systematic Reviews

Previous studies on *Dioscorea* have primarily focused on metabolic disorders, particularly blood glucose regulation in diabetes. For example, Sun et al. [39] systematically reviewed randomized controlled trials (RCTs) involving multi-herbal prescriptions containing *Dioscorea*, reporting beneficial effects for patients with T2DM, such as improved glycemic and lipid profiles, reduced insulin resistance, and fewer adverse events. Alharazi et al. [40] conducted a systematic review focusing on *Dioscorea* or its extract as a monotherapy in animal models, consistently showing reductions in fasting blood glucose and improved insulin sensitivity. However, those studies were limited to diabetes-related outcomes and did not comprehensively assess the broader gastrointestinal effects of *Dioscorea*. Moreover, the inclusion of multi-herbal formulas in previous reviews makes it difficult to isolate the individual effects of *Dioscorea*. In contrast, this review is the first to focus specifically on the physiological effects of *Dioscorea* as a single herb across various aspects of gastrointestinal health.

### 4.4. Pharmacokinetic Considerations

Pharmacokinetic aspects of bioactive compounds derived from *Dioscorea* are of particular importance when evaluating their therapeutic potential and translational relevance. Key constituents that have received repeated attention in experimental and clinical studies include diosgenin, dioscin, allantoin, polysaccharides, anthocyanins, and polyphenols. Diosgenin, a steroidal sapogenin, is known for its poor solubility in water and consequently limited oral bioavailability. Nevertheless, several reports indicate that diosgenin can undergo microbial transformation in the intestine, yielding more absorbable metabolites. Such microbiota-dependent conversion may, at least in part, explain the biological activity attributed to this compound [41,42,43,44]. Distribution analyses further suggest that diosgenin and its metabolites preferentially accumulate in the liver and intestine, consistent with their reported effects on lipid regulation and mucosal protection [25].

Dioscin, the glycosidic precursor of diosgenin, has greater polarity and solubility but is rapidly hydrolyzed in the gastrointestinal tract to release diosgenin. This interconversion highlights the role of intestinal enzymatic activity in determining systemic exposure [42]. Anthocyanins isolated from *Dioscorea alata* show a different challenge: they are chemically unstable at neutral pH and prone to rapid intestinal degradation, which limits their oral availability. Even so, a fraction can reach the colon where microbial fermentation generates phenolic metabolites that retain significant biological activity, including the anti-inflammatory properties demonstrated in models of colitis [45].

Polysaccharides such as *Dioscorea opposita polysaccharides* (DOP) follow yet another route. They are only minimally absorbed in intact form but act through prebiotic fermentation within the colon. This process leads to the production of SCFAs, which exert systemic effects on metabolism and immune regulation once absorbed into the circulation [46]. In contrast, allantoin appears to be readily absorbed and distributed, with a relatively fast renal clearance, which aligns with its reported antioxidant and mucosal-protective actions [47].

In addition, polyphenolic compounds from *Dioscorea* species represent another important class of bioactive metabolites. These molecules generally exhibit higher polarity, allowing partial absorption in the gastrointestinal tract, while a substantial fraction undergoes microbial metabolism to yield phenolic acids. Both parent compounds and their metabolites have been consistently associated with strong antioxidant properties, contributing to the attenuation of oxidative stress and the protection of intestinal mucosa. Such antioxidant activity not only complements the anti-inflammatory effects observed with other *Dioscorea*-derived constituents but also provides systemic benefits through modulation of redox-sensitive signaling pathways [48,49,50].

Taken together, these observations indicate that the pharmacokinetic profiles of *Dioscorea*-derived compounds are strongly influenced by both their chemical structures and their interaction with the gut microbiota. Lipophilic molecules such as diosgenin have limited direct absorption but may gain functional relevance through microbial metabolism, whereas hydrophilic compounds, including polysaccharides and allantoin, primarily exert local effects in the gastrointestinal tract or act through their fermentation-derived metabolites [49]. Any assessment of yam-derived interventions in vivo should therefore account for this dual contribution of direct systemic exposure and indirect microbiota-mediated pathways.

### 4.5. Part-Specific Mechanisms of Dioscorea-Derived Interventions: Microbiota Modulation and Cytoprotective Pathways

When studies were grouped by *Dioscorea* species and the plant part used, several consistent and part-dependent patterns emerged. Interventions derived from *Dioscorea opposita* tubers—including powders, resistant starch–rich preparations, polysaccharide fractions, and fermented foods—recurrently demonstrated prebiotic and fermentative effects [21]. Across rodent and human models, these tuber-based interventions promoted the expansion of beneficial taxa such as *Bifidobacterium* and *Lactobacillus*, increased short-chain fatty acid production (particularly butyrate), and enhanced intestinal barrier integrity as evidenced by improved tight-junction protein expression in animals [23,39] and reduced serum DAO and D-lactate in clinical studies [12]. At the same time, they consistently attenuated pro-inflammatory cytokines including TNF-α, IL-6, and IL-1β, and improved functional outcomes in models of constipation, diarrhea, and post-operative recovery [12,20]. Taken together, these findings indicate that *D. opposita* tuber preparations exert their beneficial actions predominantly through a microbiota–SCFA–barrier axis that appears robust and reproducible across species.

By contrast, *Dioscorea opposita* rhizome extracts exhibited a distinct profile. In ethanol-induced gastric lesion and related injury models, rhizome preparations consistently reduced ulcer indices and histopathological damage [14,15,18,37]. These protective effects were accompanied by elevated antioxidant enzyme activities (SOD and GPx), decreased lipid peroxidation (MDA), and downregulation of NF-κB–mediated inflammatory pathways, alongside upregulation of mucosal repair mediators such as EGF, VEGF, and TGF-β1 [18]. The rhizome therefore appears to act more directly on cyto-protection and tissue repair, rather than through microbial fermentation.

A similar pattern of differential activity was evident in *Dioscorea batatas*. Here, comparative studies revealed that the peel fraction provided stronger gastroprotective effects than the flesh [16]. Peel extracts more effectively suppressed gastric ulceration, enhanced antioxidant defenses (higher SOD activity and PGE_2_ levels), and reduced oxidative stress markers such as MDA. These differences are consistent with the higher phenolic and saponin content concentrated in the peel. Notably, anti-inflammatory effects of *Dioscorea batatas* peel extended beyond gastric models, improving histological outcomes and lowering cytokines in systemic inflammatory conditions such as pancreatitis [29].

For *Dioscorea alata*, both resistant starch fractions and anthocyanin-rich extracts demonstrated dual mechanisms of benefit. Resistant starches increased SCFA levels, particularly butyrate, and improved intestinal barrier function [17], while anthocyanins, despite their relatively low oral bioavailability, were metabolized by colonic microbiota into phenolic derivatives with significant anti-inflammatory activity [25]. In models of ethanol-induced gastric injury and TNBS-induced colitis, these tuber-derived preparations consistently attenuated inflammatory cytokines and enhanced tight-junction expression, supporting a synergistic mechanism that combines microbiota-dependent fermentation with antioxidant and anti-inflammatory pathways.

Taken together, these grouped findings suggest two complementary mechanistic axes: tuber-derived polysaccharides and resistant starches primarily modulate the gut microbiota and SCFA production to strengthen barrier integrity [12,13,22], while rhizome and peel-derived fractions provide rapid cytoprotective and antioxidant signaling that defends against acute injury [14,15,16,17,18,32,37]. This duality is reflected in the translational evidence, where tuber-based interventions improved barrier biomarkers and microbial ecology in human studies [12,13], whereas peel and rhizome extracts showed marked protective efficacy in acute injury and oxidative stress models [14,15,16,18,37].

### 4.6. Strengths, Limitations, and Future Directions

This review is the first to systematically analyze the multifaceted effects of *Dioscorea Rhizoma* on gastrointestinal health using both human and experimental studies. A total of 25 in vivo studies were categorized by disease type and mechanism, providing a comprehensive summary not previously available in the literature. By focusing exclusively on *Dioscorea Rhizoma* as a single agent, this study also isolates the herb’s intrinsic effects, distinguishing it from prior analyses involving multi-herbal formulations.

However, only two clinical studies were included, both with limited sample sizes and one using a non-randomized, uncontrolled design. The lack of high-quality RCTs assessing the isolated effects of *Dioscorea Rhizoma* on gastrointestinal outcomes is a significant limitation. Another limitation is considerable heterogeneity regarding plant species, plant parts, extraction processes, and dosage regimens across existing studies, which hinders reproducibility and complicates cross-study comparisons.

Future research should include large-scale, well-designed RCTs with standardized interventions, dosage protocols, and validated biomarkers to better assess the clinical utility of *Dioscorea Rhizoma* in gastrointestinal health.

## 5. Conclusions

*Dioscorea Rhizoma* may be effective in improving gastrointestinal function and holds potential for broader application in global integrative healthcare. Findings from both human and animal studies suggest beneficial roles in mucosal protection, inflammation control, motility regulation, and modulation of gut microbiota. Nevertheless, further clinical trials employing rigorous methodology and standardization of preparations are necessary to confirm these effects and support its routine use in gastrointestinal practice.

## Figures and Tables

**Table 1:** Search strategy used in PubMed.

Search Number	Search Items
#1	“dyspepsia” OR “indigestion” OR “digestion” OR “gastro” OR “epigastric” OR “stomach” OR “gastrointestinal” OR “gastr” OR “intestin” OR “colon” OR “bowel” OR “colitis” OR “Crohn”
#2	“yam” OR “dioscorea”
#3	#1 and #2

**Table 2:** Selection criteria for this review.

Items	Inclusion	Exclusion
Criteria for herbal medicines	*Dioscoreae Rhizoma* (yam)	Single-compound extracts; multi-herbal prescriptions; herbal interventions using *Dioscorea* species other than *Dioscoreae Rhizoma*
Criteria for studies	Randomized controlled trials and in vivo studies related to gastrointestinal function (e.g., gastrointestinal motility, inflammation, mucosal protection, or microbiota modulation)	Case reports; case series; in vitro studies; reviews; studies not assessing GI-related outcomes

**Table 3:** Characteristics of the included data of human studies.

Species	Plant Part Used	Herbal Intervention	Patient Inclusion Criteria	Total (*n*) (Treatment/ Control)	Control Intervention	Treatment Period	Primary Outcome	Mechanisms	Efficacy	Adverse Events (*n*) (Treatment/Control)	Reference Number
*Dioscorea opposita* Thunb. (Chinese yam)	Rhizome (yam porridge)	Yam gruel	T2DM patients post colorectal-cancer surgery	92 (46/46)	Standard care	3 weeks	↓ Wound healing time, ↓ Infection rate, ↓ Glucose (FBS, 2 h)	Glycemic regulation, enhanced recovery	Improved healing and glycemic control	Not reported	[12]
*Dioscorea alata* (Indonesian yam)	Rhizome powder (functional snack)	Yam-based high-fiber snack	Obese individuals	10 (10/0)	None	Not specified	↑ *Bifidobacterium*, ↑ *C. coccoides–E. rectale*	Prebiotic modulation of gut flora	Positive modulation of gut microbiota	Not reported	[13]

Abbreviations: ↑: significantly increased, ↓: significantly decreased, T2DM: type 2 diabetes mellitus, FBS: Fasting Blood Sugar.

**Table 4:** Characteristics of the included data of animal studies.

Species	Plant Part Used	Animal	Treatment	Disease Model	Positive Control	Admini-stration Method	Dosage	Treatment Duration	Mechanisms	Main Outcome	Efficacy	Reference Number
*Dioscorea opposita* (Chinese yam)	Rhizome extract	SD rats	*Rhizoma Dioscoreae* extract	Ethanol-induced gastric lesion	None	Oral	200 mg/kg BW/day	14 days	↑ SOD, ↑ GPx, ↑ Antioxidant [Sample: Gastric tissue homogenates (SOD, GPx)]	↓ Lesions, ↑ Stomach weight	Gastric protection	[14]
*Dioscorea opposita*	Rhizome (powder)	SD rats	Yam powder	Cysteamine-induced ulcer	Cysteamine-HCl	Oral	200 mg/kg BW/day	Single dose	↓ Ulcer, ↓ COX-2, iNOS, cytokines, ↑ CA IX/XIV [Sample: Gastric tissue (COX-2, iNOS, CA-IX/XIV)]	↓ Ulcer index	Gastric protection	[15]
*Dioscorea batatas*	Flesh and peel (EtOH extract)	SD rats	DBD Flesh and Peel (Water or Ethanol)	Ethanol-induced acute gastric ulcer	None	Oral	100, 200 mg/kg BW	Single dose	↑ SOD, ↑ PGE2, ↑ COX-2, ↓ Oxidative stress [Sample: Gastric tissue + Serum (MDA, SOD, PGE_2_, COX-2)]	↓ Gastric lesions	Gastric protection	[16]
*D. alata* (winged yam)	Resistant starch type 2	Kunming mice	RS2	Ethanol-induced gastric ulcer	Ethanol	Oral	0.64, 3.2, 6.4 g/kg BW/day	7 days	↓ ULI, ↓ MDA, ↑ SOD [Sample: Gastric tissue + Serum (MDA, SOD, histology)]	↓ Gastric damage	Gastric protection	[17]
*D. opposita*	Polysaccharide (CIYP)	BALB/c mice	CIYP	Ethanol-induced GML	None	Oral	200, 400 mg/kg BW/day	15 days	↑ MAPK signaling, ↓ Inflammation [Sample: Gastric mucosa (lesion index, NO, PGE_2_, EGF, SOD, MDA)]	↓ Gastric damage	Gastric protection	[37]
*D. opposita*	Water extract (CYW)	ICR mice	CYW	Ethanol-induced gastric injury	10 mL/kg ethanol	Oral	0.31, 0.63, 3.14 g/kg BW/day	14 days	↓ TNF-α, IL-6, IL-1β; ↑ SOD, CAT, GPx, Bcl-2/Bax, VEGF, TGF-β1 [Sample: Serum + Gastric tissue (cytokines, SOD, CAT, GPx; Bcl-2/Bax, VEGF, TGF-β1)]	↓ Ulcer index, ↓ Inflammation, ↑ Histology, ↑ Growth factors	Gastric protection	[18]
Chinese yam (NR exact species)	Ethanol extract	SD rats	Chinese yam ethanol extract	Normal (GI motility test)	None	Oral	100, 200 mg/kg BW/day	6 weeks	↓ Gastric acid, ↑ Motility, ↑ Lactose-fermenters, ↓ Glucose/lipids [Sample: Serum, feces, gastric/colonic tissue (GI transit, microflora, glucose/lipids)]	↑ Motility, ↑ Feces, ↓ Glucose	GI improvement	[19]
NR	Yam yogurt (functional food)	SD rats	Yam yogurt	Loperamide-induced constipation	Loperamide	Oral	5 g/kg BW/day	5 days	↑ Goblet cell, ↑ Mucin, ↑ Ki-67 [Sample: Feces + Colonic tissue (fecal moisture, goblet cells, Ki-67, mucin)]	↑ Fecal moisture, ↑ Evacuation	Laxative effect	[20]
*D. alata* (red/black yam)	Whole rhizome (diet)	SD rats	RY/BY	Normal physiology	None	Diet	5% *w*/*w*	3 weeks	↑ SCFAs, ↓ non-HDL cholesterol, ↓ hepatic MTP mRNA [Sample: Serum + Cecal contents + Feces (lipids, SCFAs, bile acids, hepatic mRNA)]	↓ Cholesterol, ↑ SCFA	Lipid-lowering	[21]
	Raw yam (diet)	BALB/c mice	Raw yam	Bowel modulation	None	Diet	10% *w*/*w*	21 days	↑ Fecal moisture, ↓ pH, ↑ SCFAs, ↑ crypt depth, ↑ bifidobacteria [Sample: Feces + Cecal contents + Colonic tissue (SCFAs, microbiota, crypt depth, TJs)]	↑ Microbiota, ↑ Barrier	Prebiotic effect	[22]
Chinese/Japanese yam (*D. opposita*/*D. japonica*)	Dietary rhizome	BALB/c mice	Chinese/Japanese yam	LPS-induced damage	None	Diet-fed	5% *w*/*w*	4–8 weeks	↑ SOD, ↓ MDA, ↑ Sucrase, ↓ C. perfringens [Sample: Cecal contents + Intestinal tissue (SOD, sucrase, MDA, microbiota, histology)]	↑ Gut enzymes, ↑ Microbiota	Antioxidant, microbiota modulation	[23]
*Dioscorea bulbifera*	Ethanol extract	C57BL/6 mice	DB ethanol extract	TNBS-induced colitis	5-ASA	Intra-rectal	50–100 mg/kg BW	Not specified	↑ Antioxidants, ↓ Cancer cell proliferation [Sample: Colonic tissue (MDA, SOD, histology, cancer markers)]	↓ Oxidative stress, ↓ SGC-7901	Antioxidant, anticancer	[24]
*D. alata* L.	Anthocyanins	C57BL/6 mice	DACNs	TNBS-induced colitis	5-ASA	Intra-rectal	100 mg/kg BW	7 days	↓ TNF-α, IFN-γ, iNOS, ↑ Tight junctions [Sample: Colonic tissue (cytokines, iNOS; ZO-1/occludin; histology)]	↓ Inflammation, ↑ Histology	Anti-colitic effect	[25]
Chinese yam (NR exact species)	Polysaccharide + inulin (CP)	SD rats	CP (CYP + inulin)	TNBS-induced colitis	None	Oral	200 mg/kg BW/day	16 days	↑ SCFA, ↓ MPO, ↑ Lactic bacteria [Sample: Feces + Colonic tissue (DAI, MPO, SCFAs, histology)]	↓ DAI, ↑ Colon morphology	Colitis improvement	[26]
N/A (bioactive component)	Diosgenin	BALB/c mice	Diosgenin	OVA-induced food allergy	None	Oral	50, 100 mg/kg BW/day	13 days	↑ Foxp3, IFN-γ, IL-10, Th1 chemokines [Sample: Spleen + Serum + Splenocytes (cytokines, Treg markers, NK)]	↑ Treg, ↓ Allergic inflammation	Immunomodulation	[27]
*Dioscorea opposita*	Rhizome suspension	BALB/c mice	Yam suspension	AAD	Ampicillin	Oral	4.28, 8.56, 25.68 g/kg BW/day	10 days	↑ SCFAs, ↑ gut microbiota diversity [Sample: Feces (SCFAs; 16S microbiota)]	↓ Diarrhea	Gut recovery	[28]
*Dioscorea opposita*	Peel extract	Common carp	Yam peel	Healthy feeding trial	None	Feed	5% *w*/*w*	8 weeks	↑ SOD, CAT, LZM, ↑ ZO-1, ↑ Occludin, ↑ SCFAs, ↑ Lactobacillus, ↓ Enterobacteriaceae, ↑ IL-1β, TGF-β, NF-κB [Sample: Intestinal tissue + Feces (TJ proteins, SOD, CAT, lysozyme; SCFAs; microbiota)]	↑ Tight junction, ↑ Immunity	Intestinal barrier protection	[29]
Yam extract (species NR)	Rhizome extract	Rainbow trout	Yam extract	Healthy model	None	Feed	0.1%, 0.2%, 0.4% *w*/*w*	56 days	↑ Enzymes, ↑ IL-2, IL-6, TNF-α, C4, ↑ HSPs, ↑ GPx1, ↑ Bifidobacterium, ↑ Lactobacillus [Sample: Serum + Intestinal microbiota (cytokines, antioxidative enzymes, HSPs)]	↑ Gut immunity, ↑ Microbiota	Immunostimulatory effect	[30]
NR	*Dioscorea*-based preparation (SCYP)	BALB/c mice	SCYP	Cy-induced immunosuppression	None	Oral	50, 100, 200 mg/kg BW/day	7 days	↑ SCFAs, ↑ Lactobacillus, ↑ Akkermansia, ↓ Proteobacteria [Sample: Feces + Serum + Intestine (16S microbiota; digestive enzymes; immune indices)]	↑ Gut diversity, ↑ Digestive enzymes	Gut balance restoration	[38]
*Dioscorea bulbifera*	DB extract	C57BL/6 mice	DB extract	Caerulein-induced pancreatitis	None	Oral	250 mg/kg BW	6 h	↓ Lipase, ↓ IL-6, ↓ TNF-α, No change in IL-1β [Sample: Serum + Pancreatic tissue (lipase, amylase; cytokines; histology)]	↓ Pancreas index, ↓ Damage	Anti-inflammatory	[32]
NR	Polysaccharide	ICR mice	YP	CTX-induced immunosuppression	CTX	Oral	400 mg/kg BW/day	28 days	↑ Thymus index, ↑ CD4^+^/CD8^+^, ↑ IgA/IgG/IgM, ↑ ZO-1, ↑ Occludin, ↑ SCFAs, ↑ tryptophan [Sample: Serum + Colonic tissue (IgA/IgG/IgM; ZO-1/occludin; SCFAs; metabolites)]	Immune recovery, ↑ Microbiota diversity	Immunomodulation	[33]
NR	Powder (dietary)	BALB/c mice	yam powder	DMH-induced ACF	None	Diet	5% *w*/*w*	8 weeks	↑ Apoptosis-related genes [Sample: Colonic tissue (ACF count; apoptosis-related genes)]	↓ ACF	Anti- tumorigenic	[34]
Chinese yam (NR exact species)	Rhizome + leaf juice (Aojiru)	BALB/c mice	Nagaimo/Aojiru	DMH-induced ACF	None	Diet	10% *w*/*w*	4 weeks	↑ Apoptosis [Sample: Colonic tissue (ACF; apoptosis markers)]	↓ ACF	Colon protection	[31]
NR	*Dioscorea*-based diet	F344 rats	Yam diet	AOM-induced colon cancer	AOM	Oral	10% *w*/*w*	10 weeks	↓ ACF, ↑ GSH, GPx, CAT, Cu/Zn-SOD, ↓ NF-κB, COX-2, TNF-α, IL-1β [Sample: Colonic tissue (ACF; antioxidant enzymes; inflammatory mediators)]	↓ Colon tumor markers	Anti-carcinogenic	[35]
*Dioscorea* opposite waste (DOW)	Diet adding DOW	weaned lambs	Diet adding DOW	Healthy model	Diet without DOW	Diet	10, 15, 20% DOW	62 days	↑ AST, ↑ ALP, ↑ IgA, ↑ IgM, ↑ IgG, ↑ GPx, ↑ SOD, ↓ IL-1β, ↓ IL-6, ↓ TNF-α, ↓ MDA, ↑ *Ruminococcaceae*_bacterium, ↑ *Clostridiales*_bacterium [Sample: blood plsama, rumen fluid]	↑ IgA, ↑ IgM, ↑ IgG, ↑ GPx	Antioxidant, Immunomodulation, gut micorbiota modulation	[36]

Abbreviations: ↑: significantly increased, ↓: significantly decreased, AAD: antibiotic-associated diarrhea; ACF: aberrant crypt foci; AOM: azoxymethane; ALP: alkaline phosphatase; AST: aspartate aminotransferase; Bax: Bcl-2-associated X protein; Bcl-2: B-cell lymphoma 2; BY: boiled yam; CA IX/XIV: carbonic anhydrase isoforms IX and XIV; CAT: catalase; CIYP: Chinese iron yam polysaccharide; COX-2: cyclooxygenase-2; CP: compound polysaccharide (yam + inulin); CTX: cyclophosphamide; CYP: Chinese yam polysaccharide; CYW: compound yam water extract; DACNs: *Dioscorea alata* L. anthocyanins; DAI: disease activity index; DB: *Dioscorea bulbifera*; DBD: *Dioscorea batatas* Decne; DMH: 1,2-dimethylhydrazine; DOW: *Dioscorea* opposite waste; GML: gastric mucosal lesion; GPx: glutathione peroxidase; GSH: glutathione; HDL-C: high-density lipoprotein cholesterol; IFN-γ: interferon-gamma; IgA/IgG/IgM: immunoglobulin A/G/M; IL-1β: interleukin-1 beta; IL-10: interleukin-10; IL-6: interleukin-6; Ki-67: proliferation marker protein Ki-67; L. acidophilus: *Lactobacillus acidophilus*; LPS: lipopolysaccharide; LZM: lysozyme; MDA: malondialdehyde; MPO: myeloperoxidase; MTP: microsomal triglyceride transfer protein; NF-κB: nuclear factor kappa-light-chain-enhancer of activated B cells; Occludin: tight junction integral membrane protein occludin; OVA: ovalbumin; PGE2: prostaglandin E2; RS2: resistant starch type 2; RY: raw yam; SCFA: short-chain fatty acids; SCYP: steamed Chinese yam polysaccharide; SOD: superoxide dismutase; TNF-α: tumor necrosis factor-alpha; TGF-β: transforming growth factor-beta; YP: yam polysaccharide; ZO-1: zonula occludens-1.

## Data Availability

The data presented in this review are available on request from the corresponding author due to privacy.

## Supplementary Materials

The following supporting information can be downloaded at: https://www.mdpi.com/article/10.3390/nu17182943/s1, Table S1: Summary of SYRCLE-based risk of bias.

## Abbreviations

The following abbreviations are used in this manuscript:

AAD: antibiotic-associated diarrhea; ACF: aberrant crypt foci; ALP: alkaline phosphatase; AMED: Allied and Complementary Medicine Database; AOM: azoxymethane; AST: aspartate aminotransferase; Bax: Bcl-2-associated X protein; Bcl-2: B-cell lymphoma 2; BY: boiled yam; CA IX/XIV: carbonic anhydrase isoforms IX and XIV; CAT: catalase; CiNii: Citation Information by NII; CIYP: Chinese iron yam polysaccharide; CNKI: China National Knowledge Infrastructure; COX-2: cyclooxygenase-2; CP: compound polysaccharide (yam + inulin); CRC: colorectal cancer; CTX: cyclophosphamide; CYP: Chinese yam polysaccharide; CYW: compound yam water extract; DACNs: *Dioscorea alata* L. anthocyanins; DAI: disease activity index; DAO: serum diamine oxidase; DB: *Dioscorea bulbifera*; DBD: *Dioscorea batatas* Decne; DMH: 1,2-dimethylhydrazine; DOP: *Dioscorea opposita* polysaccharides; DOW: *Dioscorea* opposite waste; FBG: fasting blood glucose; FBS: fasting blood sugar; GC: gas chromatography; GI: gastrointestinal; GML: gastric mucosal lesion; GPx: glutathione peroxidase; GSH: glutathione; HDL: high-density lipoprotein; HDL-C: high-density lipoprotein-cholesterol; HOMA-IR: homeostatic model assessment for insulin resistance; IFN: Interferon; Ig: immunoglobulin; IL: interleukin; iNOS: inducible nitric oxide synthase; *L. acidophilus*: *Lactobacillus acidophilus*; LPS: lipopolysaccharide; LZM: lysozyme; MDA: malondialdehyde; MPO: myeloperoxidase; MTP: microsomal triglyceride transfer protein; N/A: not applicable; NF-κB: nuclear factor kappa-light-chain-enhancer of activated B cells; NI: No information; OASIS: Oriental Medicine Advanced Searching Integrated System; OVA: ovalbumin; PGE2: prostaglandin E2; PRISMA: Preferred Reporting Items for Systematic Reviews and Meta-Analyses; RCT: randomized controlled trial; RoB: Risk of Bias; RoB 2: Cochrane Risk of Bias Tool 2.0; ROBINS-I: Risk Of Bias In Non-randomized Studies—of Interventions; ROS: Reactive Oxygen Species; RS2: resistant starch type 2; RY: raw yam; SC: Some concerns; SCFA: short-chain fatty acids; SCYP: steamed Chinese yam polysaccharide; SD: Sprague-Dawley; SOD: superoxide dismutase; SYRCLE: Systematic Review Centre for Laboratory Animal Experimentation; T2DM: type 2 diabetes mellitus; TC: total cholesterol; TGF: transforming growth factor; Th1: Type 1 T helper; TNBS: 2,4,6-Trinitrobenzenesulfonic acid; TNF: tumor necrosis factor; Treg: regulatory T cell; VEGF: vascular endothelial growth factor; YP: yam polysaccharide; ZO-1: zonula occludens-1.
