# Supplementary material for: The Effect of Dioscoreae Rhizoma on Gastrointestinal Function: A Systematic Review
Source: Nutrients. 2025 Sep 12;17(18):2943. doi: 10.3390/nu17182943 (PMC12472200; doi:10.3390/nu17182943)
Supplement: Supplementary file 1 [file nutrients-17-02943-s001.zip › nutrients-3809292-supplementary.pdf]

Supplementary Table 1. Summary of SYRCLE-based risk of bias.

Study (Author, Year) [Reference number]	D1	D2	D3	D4	D5	D6	D7	D8	D9	D10	Over-all
Jeong et al., 2009 [14]	NI	Low	NI	NI	High	NI	High	Low	Low	Low	High
Park et al., 2013 [15]	SC	Low	NI	NI	NI	NI	High	Low	Low	Low	High
Byeon et al., 2018 [16]	Low	Low	NI	NI	SC	NI	SC	Low	Low	Low	SC
Mao et al., 2018 [17]	SC	Low	NI	NI	NI	NI	SC	Low	High	Low	High
Guo et al., 2020 [18]	Low	Low	Low	Low	SC	Low	SC	Low	Low	Low	Low
Xie et al., 2024 [19]	Low	Low	NI	NI	NI	SC	SC	Low	SC	Low	SC
Jeon J et al., 2006 [20]	Low	Low	SC	SC	SC	SC	SC	Low	Low	SC	SC
Jeon et al., 2007 [21]	Low	SC	Low	Low	Low	SC	Low	Low	Low	Low	Low
Nishimura et al., 2011 [22]	NI	Low	NI	NI	NI	NI	NI	Low	Low	Low	SC
Wang et al., 2007 [23]	SC	Low	NI	NI	NI	NI	SC	Low	SC	Low	SC
Hsu et al., 2006 [24]	Low	Low	Low	Low	Low	Low	Low	Low	Low	Low	Low
Chen et al., 2013 [25]	NI	Low	NI	NI	NI	NI	NI	Low	Low	NI	NI
Chen et al., 2017 [26]	NI	Low	NI	NI	NI	NI	NI	Low	Low	NI	NI
Cai et al., 2019 [27]	SC	SC	NI	NI	NI	SC	SC	Low	SC	Low	SC
Chung-Hsiung et al., 2017 [28]	Low	Low	SC	NI	NI	NI	NI	Low	Low	Low	SC
Zhang et al., 2019 [29]	SC	SC	NI	NI	NI	NI	SC	Low	SC	SC	SC
Meng et al., 2019 [30]	SC	Low	NI	SC	SC	SC	SC	Low	SC	Low	SC
Wang et al., 2020 [31]	SC	Low	NI	NI	NI	NI	SC	Low	SC	Low	SC
Huang et al., 2021 [32]	Low	Low	SC	SC	NI	SC	NI	Low	Low	Low	SC
Kweon et al., 2022 [33]	High	Low	Low	Low	Low	Low	SC	High	High	SC	High
Lu et al., 2024 [34]	SC	Low	Low	Low	Low	Low	SC	High	Low	SC	SC
Kinoshita et al., 2008 [35]	SC	SC	NI	SC	NI	SC	SC	Low	SC	SC	SC
Kinoshita et al., 2009 [36]	SC	Low	NI	NI	NI	NI	SC	Low	SC	SC	SC
Son et al., 2014 [37]	SC	Low	NI	NI	NI	NI	SC	Low	SC	Low	SC
Yang et al., 2023 [38]	SC	Low	NI	NI	NI	NI	SC	Low	Low	Low	SC

Abbreviations: SYRCLE: Systematic Review Center for Laboratory animal Experimentation; D1: Sequence generation; D2: Baseline characteristics; D3: Allocation concealment; D4: Random housing; D5: Blinding of caregivers/investigators; D6: Random outcome assessment; D7: Blinding of outcome assessor; D8: Incomplete outcome data; D9: Selective outcome reporting; D10: Other sources of bias; NI: No information; SC: Some concerns.
